# Two Simultaneous Primary Cancers: A Case Report

**DOI:** 10.7759/cureus.81999

**Published:** 2025-04-10

**Authors:** Catalina Gonzalez Aguirre, Elias Gallardo-Navarro, Antonio Castillo Magaña

**Affiliations:** 1 Oncologic Surgery, Spanish Hospital, Mexico City, MEX; 2 General Surgery, Spanish Hospital, Mexico City, MEX; 3 Head and Neck Surgery, Spanish Hospital, Mexico City, MEX

**Keywords:** head and neck neoplasms, multiple primary tumors, primary testicular lymphoma, soft tissue sarcoma, synchronous tumor

## Abstract

When a patient is diagnosed with more than one tumor in the same organ or in a different one, there may be multiple primary tumors. We present the case of a patient with an initial diagnosis of undifferentiated pleomorphic sarcoma (UPS) of the posterior region of the neck in whom an extension study was requested after surgery prior to the systemic therapy; tumor activity was identified in the left testicle, and radical orchiectomy was performed with the result of non-Hodgkin lymphoma (NHL) B cells. Multiple primary neoplasms have different histologies and different sites of origin. The interest of the case arises from the clinical presentation in which the patient is being staged due to a previously diagnosed malignant tumor, and a second tumor is detected in the positron emission tomography (PET) scan. Because of the characteristics of the first neoplasm, it is very unlikely that it could correspond to metastasis of the known primary tumor; therefore, it is suspected to be another primary tumor that requires histological confirmation.

## Introduction

Multiple primary tumors or synchronous primary cancer are defined as the coexistence of more than one primary neoplasm in different organs, or the coexistence of two or more primary neoplasms of different cell types in the same organ, these multiple primary neoplasms have different histology and site of origin and these cancers develop independently of each other, risk factors associated with this condition such as genetic burden such as BRCA mutations, hormonal factors such as hormone therapy, prior exposures to cancer diagnosis and treatment, harmful agents such as alcohol and tobacco, as well as environmental influences such as areas with increased radon exposure and workers exposed to asbestos associated with mesothelioma, viruses such as Epstein-Barr virus and human papillomavirus are related to cancers [1,2]. The two most common definitions currently in use are those provided by the Surveillance Epidemiology and End Results (SEER) project and the International Association of Cancer Registries (IACR) and the International Agency for Research on Cancer (IARC) [2]. The SEER database recommends using a two-month period to distinguish between synchronous and metachronous multiple primaries. Rules according to the IARC suggest the registration of synchronous tumors diagnosed in an interval of less than six months or metachronous if more than six months, if arising in different sites [1-3]. The synchronous primary tumors in our patient include an undifferentiated pleomorphic sarcoma in the posterior region of the left neck and a second primary tumor, diagnosed through an extension study using positron emission tomography (PET), which was compatible with B-cell non-Hodgkin lymphoma (NHL) in the left testicle.

Sarcoma currently represents approximately 1% of tumors in the adult population. The presentation in the head and neck region is rare, particularly those in the soft tissues of the craniofacial region, the most common sites being the sinonasal tract and the facial skeleton [2-6]. It was first described by O'Brien and Stout under the name of malignant fibrous xanthoma; subsequently, undifferentiated pleomorphic sarcoma (UPS) was included under the malignant tumors of uncertain differentiation of the 2020 WHO soft tissue sarcoma classification [2, 6-8]. A UPS is a rare subtype characterized by the lack of specific immunohistochemical markers for a given lineage differentiation; its cellular heterogeneity is the key distinguishing feature, harboring tumor cells with marked cellular pleomorphism composed of different cell types with undifferentiated morphology [4, 9-11]. The most common differential diagnoses considered for UPS are lipoma, liposarcoma, angiosarcoma, angioma, leiomyosarcoma, osteosarcoma, and tumor metastases from other sites are also considered [4,10,11]. As for the second primary tumor, there are between 1% and 2% of cases of lymphoma, which involve the testicle as primary testicular NHL; in 35% of patients, the tumor involves both testes, and it usually presents as a painless testicular mass. Therefore, in most cases, the initial treatment is radical orchiectomy [8]. When two active malignancies are diagnosed simultaneously in a patient, the challenge is to develop an anticancer therapeutic strategy that addresses both types while minimizing toxicity, avoiding significant drug interactions, and reducing the overall negative impact on the patient's outcome [3, 6, 8].

Both primary tumors, head and neck sarcoma and primary testicular lymphoma, are rare neoplasms, each individually; treatment depends on the clinical stage of both neoplasms, with multidisciplinary management including radiotherapy, medical oncology, and surgery, giving an optimal management that can be individualized depending on important risk factors such as clinical stage, degree of malignancy of the tumor, and the availability of treatment, so currently the reported cases are limited. The importance of this case is the absence of common symptoms of each particular neoplasm.

## Case presentation

**Figure 1:**
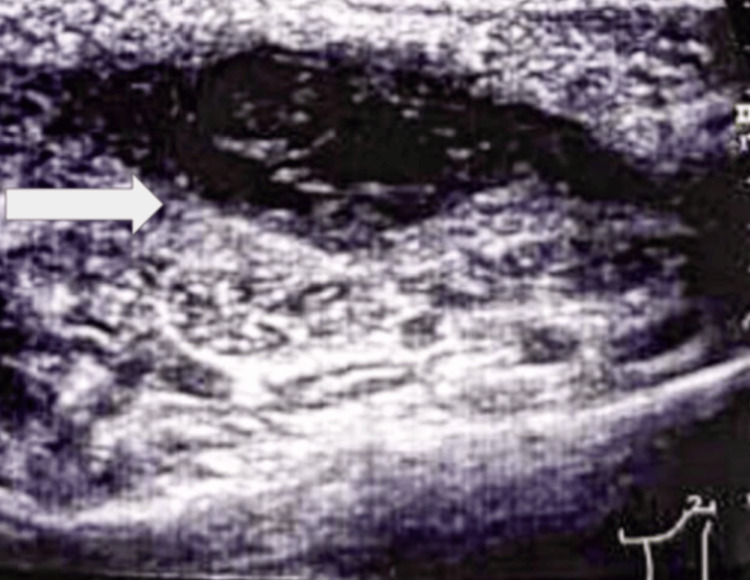
An ultrasound image of the neck (arrow) shows a space-occupying lesion of oval shape, parallel to the skin, with partially circumscribed margins, with heterogeneous echogenicity, measuring 26 x 22 x 11 mm, with a volume of 33 mm.

**Figure 2:**
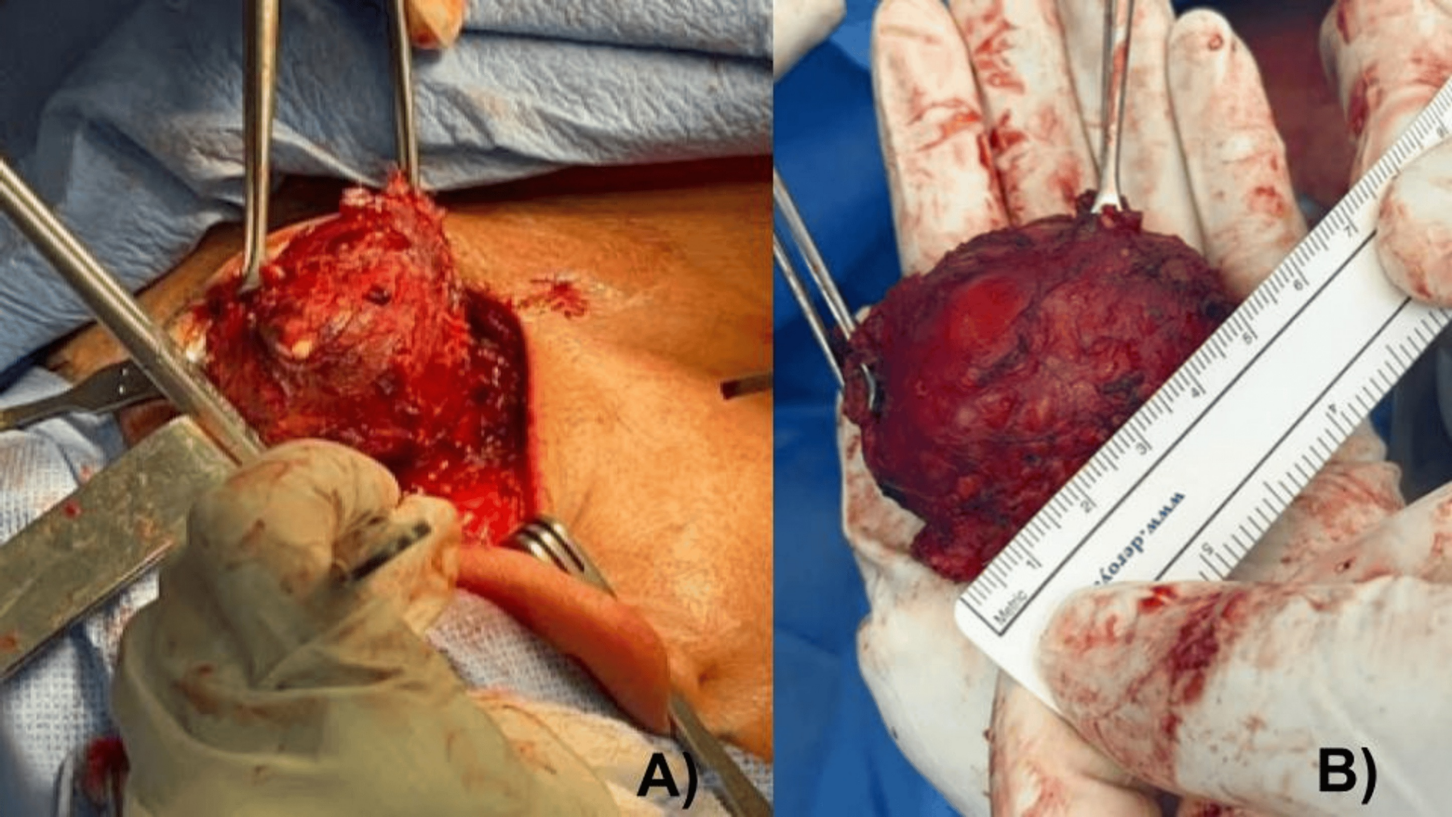
(A) Intraoperative image of tumor resection in the left posterior triangle of the neck; (B) Complete resection of the soft-tissue tumor, weighing 50 g and measuring 5.5x5.0x4.0 cm, the external surface is anfractuous and covered entirely by adipose tissue.

The patient was a 54-year-old male who consulted due to a progressive, non-painful increase in growth in the left posterior cervical region of approximately four months of evolution, with no significant personal or family pathological history and negative smoking habits and alcoholism. On physical examination, there was evidence of a tumor in the left posterior cervical region, mobile, with regular edges and smooth texture, no sensitivity or color changes in the overlying skin, and a neck ultrasound was requested (Figure 1), where multiple nodules of inflammatory aspect were identified in the cervical VA and left VB lymph node level predominantly. In addition, a space-occupying lesion of oval shape was visualized, parallel to the skin at the same level, with partially circumscribed margins, with heterogeneous echogenicity, measuring 26 x 22 x 11 mm, a volume of 33 mm, and increased peripheral vascularity. Blood biometry, red blood cell, white blood cell, and platelet levels, and coagulation tests were within normal parameters; no tumor markers were detected. With all this information, the patient was scheduled for soft tissue tumor resection of the neck (Figure 2). Histopathological analysis confirmed a diagnosis of undifferentiated pleomorphic sarcoma.

**Figure 3:**
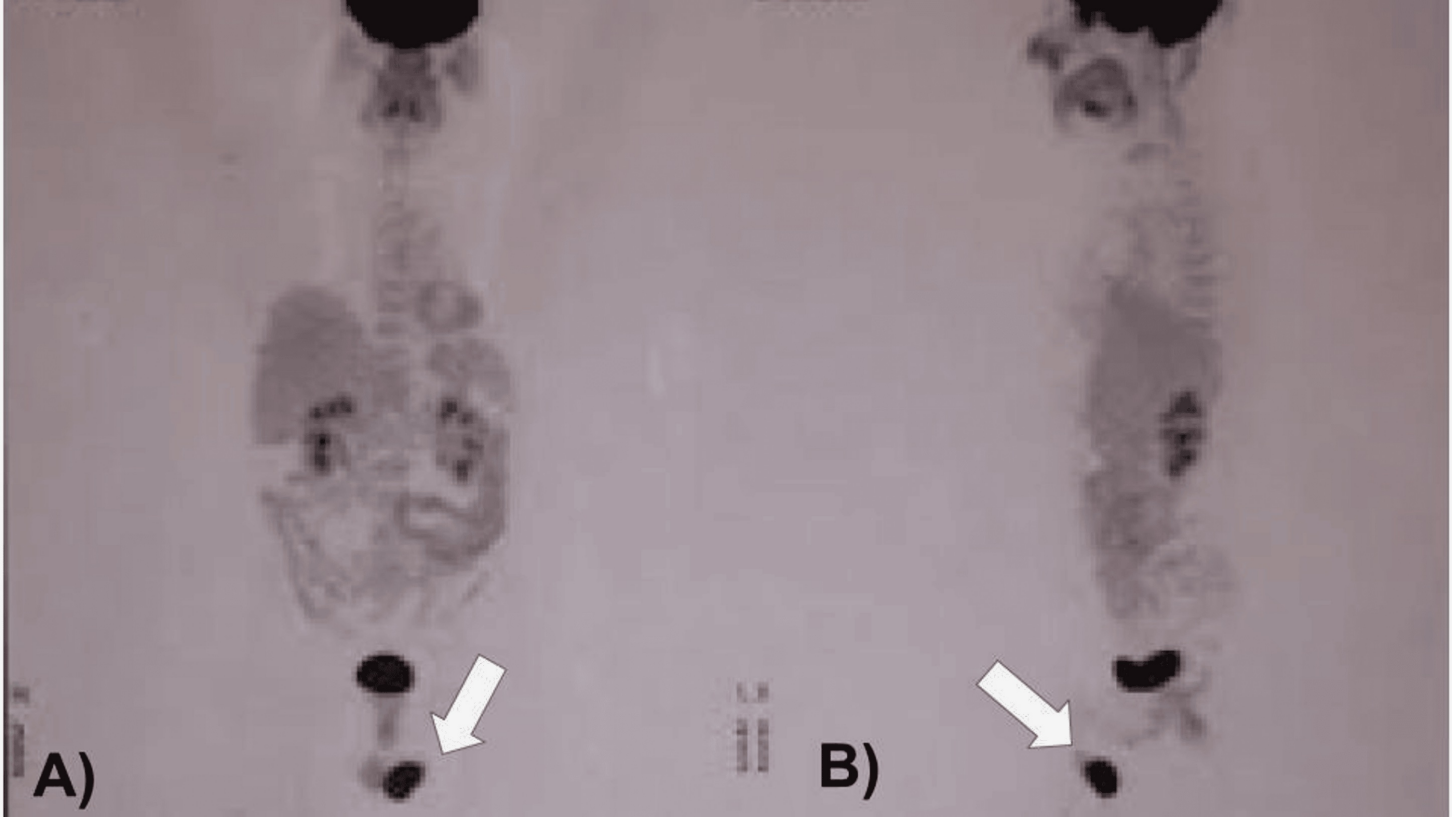
Body PET-CT with 18-fluorodeoxyglucose (FDG): (A) coronal and (B) sagittal image, with a white arrow in both images, show a solid lesion with lobulated edges in the left testicle, measuring 38 mm at the major axis, which is associated with focal hypermetabolism with a maximum standardized uptake (SUVmax) value of 32.6; no other alteration is observed.

**Figure 4:**
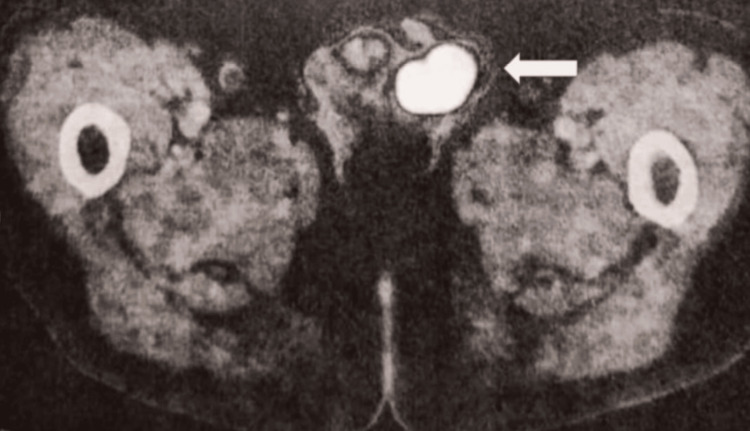
Body PET-CT with 18-fluorodeoxyglucose (FDG) axial image (arrow) shows a solid lesion in the left testicle, measuring 38 mm on the major axis.

**Figure 5:**
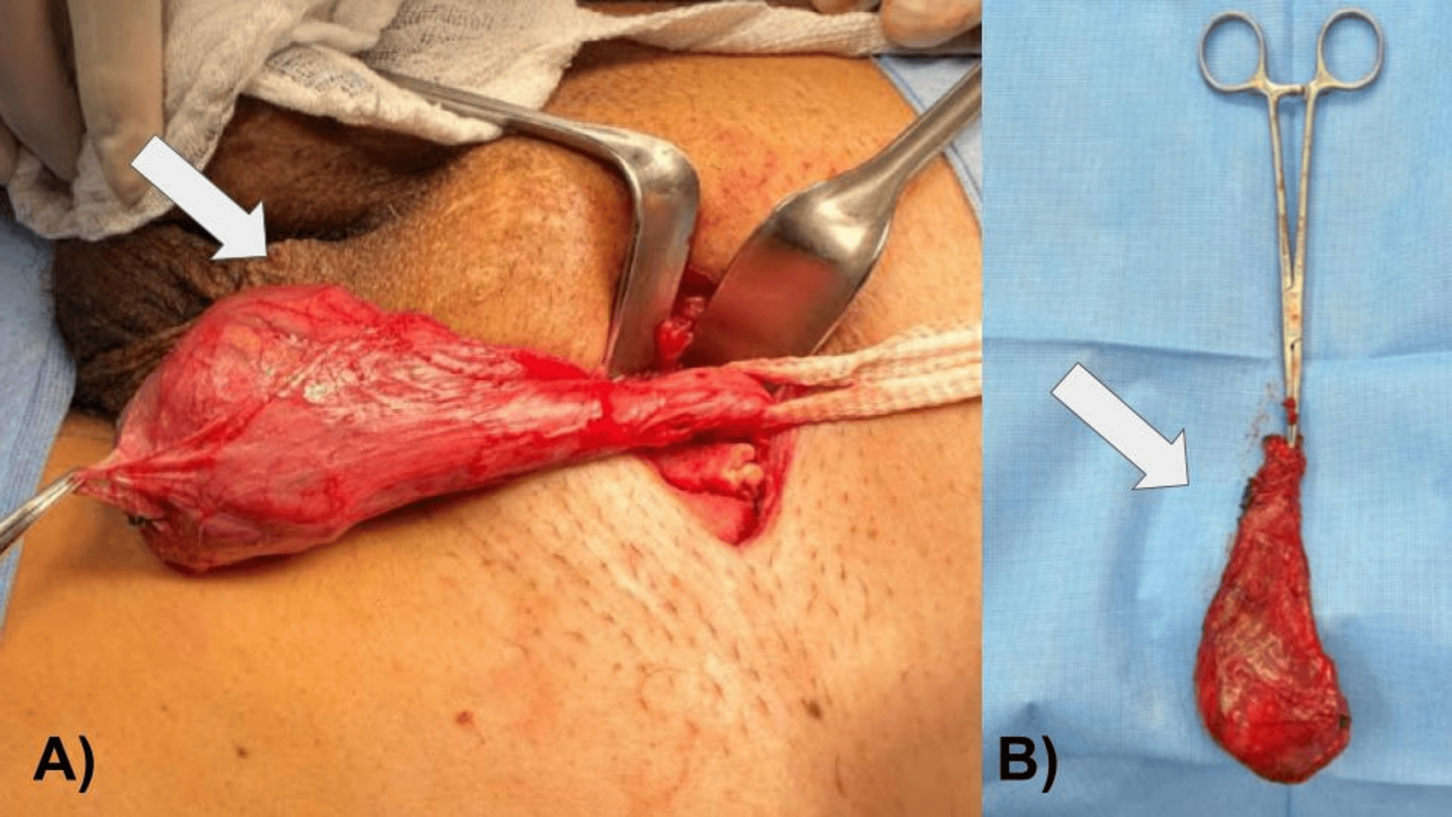
(A) Intraoperative image of left inguinal radical orchiectomy; (B) Complete resection of the left testicle and spermatic cord, weighing 64.5 g, the testicle measures 6.0 x 4.0 x 3.5 cm, the spermatic cord is 6.5 x 2.0 cm, the tunica vaginalis is grayish and anfractuous.

The staging study with PET was requested (Figures 3-4), with the result of a left testicular lesion associated with focal hypermetabolism compatible with neoplastic activity, a maximum standardized uptake value (SUVmax) of 32.6. With the result of the PET, a more thorough physical genital examination was performed, and a solid palpable tumor was identified in the left testicle with negative inguinal nodules, no pain, and no glands were palpated in any other clinically positive region; the patient also denied weight loss, night sweats, lack of appetite, or any other additional symptoms. Tumor markers were requested; alpha-fetoprotein, beta-human chorionic gonadotropin (hCG), and lactate dehydrogenase were found to be in the normal range. A left radical orchiectomy was performed (Figure 5) with a histopathology result of non-Hodgkin B-cell lymphoma. With this result, treatment started with adjuvant chemotherapy administered with six cycles of chemotherapy with cyclophosphamide, doxorubicin, vincristine, and prednisone with rituximab (R-CHOP) and one intrathecal chemotherapy with methotrexate for the treatment of non-Hodgkin B-cell lymphoma by the medical oncology department, then 30 sessions of radiotherapy with 2 Gy in a single fraction per day for the treatment of pleomorphic sarcoma of the neck, and concluded with 15 sessions of radiotherapy with 30 Gy in a single fraction per day to the contralateral testicle. Currently, eight months after concluding treatment and with complete remission of both primary tumors in different anatomical regions, the patient continues surveillance with a negative PET scan at the moment.

## Discussion

The management in relation to the type of tumor, the degree of malignancy, and the clinical stage are the most important factors for a better outcome for the patient. As for the clinical presentation of sarcoma, the duration of symptoms can vary, usually presenting as asymptomatic, fast-growing tumors without superficial skin abnormalities. The clinical presentation of testicular lymphoma is usually seen as a painless scrotal lump not separate from the testis, with no preference for laterality, although 40% of patients with primary testicular lymphoma may be accompanied by hydrocele or present as a bilateral mass in 6% to 10% of cases; this is consistent with currently reported cases [8,9]. Bacterial epididymo-orchitis, primary testicular tumor, testicular infarction, and genitourinary tuberculosis must be considered during the differential diagnosis [7,8,9,11]. In our patient, the clinical manifestation was a chronic testicular mass, which did not cause any symptoms, and an asymptomatic neck mass; the patient did not consult a doctor earlier because no pain was present in either of the tumors. The treatment of sarcomas is guided by the stage, location, size, and age of the patient. Magnetic resonance imaging (MRI) is essential for preoperative staging and surgical planning, while whole-body computed tomography (CT) or PET-CT can be used to rule out distant metastases. Currently, no standard therapeutic strategy has been established. However, due to the delicacy of the structures of the head and neck area, the possibility of performing radical surgery is limited; this is only possible in small tumors without significant invasion. A mutilating radical resection is not justified if it has no chance of being curative [2, 9-11]. A comparison of surgical and nonsurgical treatment revealed that patients who underwent surgery had higher five-year local control rates (56% vs. 40%) and higher five-year survival rates (70% vs. 40%) [12]. Currently, the established treatment of testicular lymphoma is transinguinal radical orchiectomy; adjuvant systemic chemotherapy includes six cycles of cyclophosphamide, hydroxydaunorubicin, vincristine, and prednisone (CHOP); rituximab (R-CHOP) can be added; this standard treatment was conducted in the treatment of our patient [8,9]. In the situation of a new diagnosis of cancer, staging tests are performed to assess the extent of the disease, as was performed in our patient. In addition, patients with a previous diagnosis of cancer, often over several years, follow-up tests, and examinations are performed to rule out a relapse, and in this period is where you come to observe a new tumor or known risk factors, such as association with different immunosuppression syndromes [1, 13, 14]. Patients with a history of radiotherapy have an increased risk of breast, lung, thyroid, and colorectal cancer, and chemotherapy is associated with an increased risk of secondary leukemias, lung, gastrointestinal, or bladder cancer, as well as soft tissue and bone sarcomas [3, 1, 14]. Tumor grade reflects the morphological differences between tumor tissues and normal tissue cells in terms of tissue structure and cell morphology and may reflect well the biological characteristics and metastatic potential of tumors [15,16]. Radiotherapy and chemotherapy for pleomorphic sarcoma are recommended as adjuvant therapies, and immunotherapy such as ipilimumab (anti-CTLA4), nivolumab (anti-PD1), and pembrolizumab (anti-PD1) are the current treatment modalities for this type of sarcoma; our patient did not undergo immunotherapy. [17, 18, 19]. Regarding primary testicular lymphoma, studies have identified several prognostic factors, such as age older than 70 years, advanced stage, presence of systemic symptoms, functional status, involvement of more than one extranodal site, tumor size larger than 10 cm, elevated serum levels of lactate dehydrogenase and beta (β)-2-microglobulin, as well as hypoalbuminemia, usually have a worse clinical course, and prognosis is usually poor [11, 20, 21]. The treatment of synchronous primary cancers can be complex, as each cancer must be treated according to its own characteristics and stage. Treatment decisions often involve a multidisciplinary approach, including surgery, radiation therapy, and chemotherapy, with a focus on managing both cancers simultaneously or in sequence to optimize the patient’s health. The prevalence of multiple primary tumors increases in relation to several factors, such as the increase in oncological treatments and the increase in the use of cytotoxic agents and ionizing radiation as treatment for some other conditions. The number of patients with secondary tumors may also increase with the increase in the number of patients with cancer in subsequent years. The risk of a second cancer is higher than that of the first, which may be an indication of a particular exposure, an inherited set of genes, or both. Multiple primary tumors are site-specific, and the risk varies with the anatomic location of the first primary [22]. Due to the rarity and complexity of this type of disease, accurate histopathological classification and staging of each tumor are important, as they provide the basis for individualized treatment strategies for each tumor type [23].

## Conclusions

The uniqueness of this case lies in the rarity of head and neck sarcoma, both in terms of its origin and anatomical location. Additionally, another primary tumor compatible with testicular lymphoma was found in a patient with no significant medical history and no known genetic mutations. The second tumor was incidentally discovered during the staging of the first. With this information, we can establish personalized treatment strategies to optimize patient outcomes while minimizing morbidity.
